# A single-cell transcriptomic atlas elucidates the hair cycle and apoptosis mechanisms in goat hair follicles

**DOI:** 10.3389/fcell.2025.1693637

**Published:** 2025-11-05

**Authors:** Chongyan Zhang, Feng Yang, Zhihong Liu

**Affiliations:** ^1^ Inner Mongolia Agricultural University Animal Science Department, Hohhot, China; ^2^ China Meat Research Center, Beijing Academy of Food Sciences, Beijing, China; ^3^ Inner Mongolia Key Laboratory of Sheep & Goat Genetics Breeding and Reproduction, Hohhot, China; ^4^ Key Laboratory of Mutton Sheep & Goat Genetics and Breeding, Ministry of Agriculture and Rural Affairs, Hohhot, China

**Keywords:** goat, hair follicle, single-cell transcriptomic, apoptosis, API5, matrix cells

## Abstract

To explore the molecular mechanism of hair follicle apoptosis in the goat hair cycle, single-cell transcriptome sequencing was performed on 8,214 hair follicle cells in the Anagen period, Catagen period, and Telogen period. We identified nine cell types and found that Matrix cells (MCs) enriched the Wnt signaling pathway and apoptosis-related genes. Further studies revealed the key dynamic genes (such as KRT19, API5) and regulatory factors (such as MSX2, LEF1) that determine the fate of MCs. This study mapped a high-resolution cell map of goat hair follicle apoptosis, providing new insights and diagnostic biomarkers for programmed cell death.

## 1 Introduction

A hair follicle is an accessory organ of the skin. It is developed from embryonic epithelial cells and has complex morphology and structure. It is directly involved in the formation of villi and the regulation of growth and development in cashmere goats (Plikus et al., 2015). Hair follicles undergo a regular cycle of degeneration and regeneration throughout their lives, including the growth cycle (Anagen), apoptosis-mediated degeneration phase (Catagen), and relative stationary phase (Telogen). During these three periods, the morphological structure of hair follicles will change, and each growth stage will be affected by a variety of signal regulation (Tseveenjav et al., 2020). This change is often related to the regulation of gene expression (Rahmani et al., 2014; Panteleyev, 2018; Jing et al., 2021; Shin et al., 2020). Seiberg et al.proposed that the three stages of the hair cycle are related to apoptosis (Farrier and Morgan, 1997). Apoptosis of these cells is regulated by hormones and growth factors, and none of these hormones or growth factors can maintain their activity throughout the hair follicle growth cycle. Hormones and growth factors can only work in a specific stage, in a specific part of the body, and can activate the next stage of the factors, so that the entire hair follicle cycle can operate normally. In order to maintain the vigorous growth of hair and prolong the Anagen period, it may be a feasible method to delay the arrival of Catagen and shorten the duration of Telogen. Therefore, the study on the generation of hair follicle apoptosis cycle and the mechanism of hair follicle will fundamentally lay a biological foundation for the increase of cashmere yield and quality improvement (Zheng et al., 2019).

Traditional sequencing technology takes tissue as a unit, which makes the problem of cell heterogeneity in complex tissues covered up, and the study of gene expression and its transcriptional regulation mechanism has been greatly limited. Single-cell sequencing technology can overcome (Hwang et al., 2018; Kolodziejczyk et al., 2015; Chambers et al., 2019). Therefore, in this study, to solve the problem of a lack of information on cell heterogeneity and molecular pathways determined by key cell fates during cashmere follicle development, we established a cell map during the cycle transformation of cashmere goat hair follicles. The current study provides valuable information for the identification of biomarkers by dissecting the cellular heterogeneity during hair follicle development in cashmere goats. In addition, the cell lineage inference analysis provides a comprehensive understanding of the molecular pathways that determine the fate of the major cell lineages, which has implications for future cashmere goat breeding and provides in-depth insights into the development of hair follicles.

## 2 Materials and methods

### 2.1 Experimental animals

Three female Inner Mongolian cashmere goats, aged 24 months, were employed in the study by Jinlai Animal Husbandry Technology Co., Ltd. The goat’s scapula’s wool was severed with scissors, and the exposed flesh was first cleaned with 75% alcohol and then again with iodoform. Subsequently, a 1 cm^2^ section of skin was removed using sterile scissors and preserved in PBS for future utilization. Samples were collected by the Guidelines for Experimental Animals of the Ministry of Science and Technology (Beijing, China) and were approved by the experimental animal ethics committee of Inner Mongolia Agricultural University (GB14925-2001).

### 2.2 Single-cell suspension preparation

Before being digested, skin tissues were taken from a minimum of three independent females. The back skin tissues were separated by microdissection, and the dorsal skin tissues were digested at 37 C for 30 min using a 0.25% trypsin/EDTA solution, mechanically dissociating the tissue once every 10 minutes, in order to prepare the dorsal skin single-cell suspension for single-cell RNA sequencing. The tissue was digested by trypsin at 37 C for 30 min, and then 1 mg/mL collagenase IV (Sigma, St Louis, MO, USA) was utilized. Pipetting was used to mechanically separate the skin tissues into single-cell suspensions. Before building a single cell library, the cell suspensions were filtered through a 30 μm nylon cell strainer to exclude villus debris (BD Falcon, BD Biosciences, San Jose, CA, USA).

### 2.3 Single-cell library construction and sequencing

Single-cell barcoding and library preparation were performed based on the 10x Genomics single-cell RNA sequencing platform (10x Genomics, Pleasanton, CA, USA). Briefly, the single cell suspension prepared above was immediately counted using a hemocytometer (TC20, Bio-Rad, Hercules, CA, USA), and the cell concentrations were adjusted to 1,000 cells/μl before barcoding. To barcode the single cells with 10x Barcoded gel beads, 10x Genomics Chromium Single Cell 3′ Library & Gel Bead Kit v2 (10x Genomics Inc., Pleasanton, CA, USA, 120,237) and the 10x Genomics Chromium barcoding system were used to construct 10x barcoded cDNA library following the manufacturer’s instructions. Illumina HiSeq X Ten sequencer (Illumina, San Diego, CA, USA) was used for sequencing, and pair-ended 150 bp (PE150) reads were generated for downstream analysis.

### 2.4 10X genomics scRNA-seq data processing

The CellRanger (v2.2.0) software was used for analyzing raw sequencing data according to the 10x Genomics official pipeline (https://support.10xgenomics.com/single-cell-gene expression/software/pipelines/latest/what-is-cell-ranger). Briefly, the sequencing raw base call (BCL) files were first transformed into FASTQ files with the ‘cell ranger mkfastq’ function. The generated FASTQ files were then processed with the ‘cell ranger count’ wrapped function with the ‘--force-cells = 7,000’ argument to adjust the sample size. The Cell Ranger count function used the wrapped STAR software to align the sequence to the reference genome. The output files containing gene expression matrices and barcode information of the CellRanger pipeline were then used for downstream visualization analysis. The goat ARS1 reference genome downloaded from Ensembl was used as a reference genome (https://asia.ensembl.org/Capra_hircus/Info/Index).

After the CellRanger pipeline, the Quality Control (QC) and cell clustering were analyzed with single-cell RNA seq Seurat software (v2.3.0) based on the R environment (R version: 3.5.3, https://www.r-project.org/) following the online guide (https://satijalab.org/seurat/). We used ‘filtered_gene_bc_matrices’ files generated by CellRanger as input files for Seurat. After normalization, the variable genes for each dataset were calculated for the downstream clustering assay.

To compare transcriptome profiles along three different developmental timepoints, we then merged three different datasets using the ‘RunMultiCCA’ function implemented in Seurat.

RunMultiCCA used a canonical correlation analysis to remove variation caused by the sample source. After dataset alignment, we then performed a clustering analysis on the integrated dataset based on the t-distributed Stochastic Neighbor Embedding (t-SNE) algorithm implemented in Seurat. To identify cluster-specific expressed genes, we used Seurat to implement the ‘FindAllMarkers’ function to calculate cluster markers, and the t-SNE identified cell clusters were annotated based on the previously reported canonical marker genes expression.

To subcluster cell clusters of interest for in-depth analysis and/or downstream differentiation trajectory construction, we used Seurat’s implemented ‘SubsetData’ function to extract clusters of interest.

### 2.5 Single-cell pseudotime lineage trajectory reconstruction

To interpret cell differentiation fate decisions, we used Monocle (v 2.4.0) to order single cells along pseudotime according to the official tutorial (http://cole-trapnell-lab.github.io/monocle-release/docs/#constructing-single-cell-trajectories). To perform pseudotime ordering for particular cell types, we first subclustered the interested cell type from the Seurat object, then the Monocle object was constructed using the ‘newCellDataSet’ function in Monocle. To order single cells along pseudotime, we used Seurat-identified variable genes as ordering genes to construct a single cell differentiation trajectory. The root state was set according to the cell Seurat identified cell cluster label, and the ‘BEAM’ function was used to calculate branch-specific expressed genes. To plot a branch-specific expression heatmap, we used Monocle’s implemented ‘plot_genes_branched_heatmap’ function, and genes with q-val < 1e-4 were regarded as input genes. The different gene sets were calculated according to k-means clustering.

### 2.6 Bioinformatics analysis

Gene Ontology (GO) analysis identifies the important connections between various genes and biological functions. The highly variable genes found in each cell cluster were subjected to GO enrichment analysis in this experiment using the ClusterProfiler R software tool. Furthermore, the bitr function was used to convert the symbol gene IDs into Entrez IDs. Based on the results of the significance of the difference test, the enrichment of differentially expressed genes was examined; a P value of less than 0.05 was considered significant gene enrichment.

Gene sets from Anagen, Catagen, and Telogen goat hair follicles were analyzed using Gene Set Enrichment Analysis (GSEA) software based on the greatest enrichment of differentially expressed genes (DEGs) per cell type. At the top or bottom of these arranged gene vectors was the GSEA algorithm’s test gene set. The gene list was arranged with upregulated differentially expressed genes at the top and downregulated differentially expressed genes at the bottom. The R package “heatmap” function was used to visualize the results.

To assess the differentiation status of hair follicle cells, the differentiation specificity of each cell was observed using CytoTRACE, which predicted the developmental potential and relative differentiated state of each cell. Subsequently, we imported CytoTRACE into the R package and executed the CytoTRACE function on the customized RNASeq dataset. The diminution of data dimensionality was shown using t-SNE.

To ascertain the principal transcriptional regulators involved in hair follicle apoptosis, putative transcriptional regulators were identified by single-cell regulatory network inference and clustering (SCENIC) analysis. Motif enrichment analysis was conducted for each coexpression module. According to the matrix, only the target genes enriched with motifs of transcription factors were preserved, while other genes were eliminated. Transcription factors and their direct target genes were designated as regulons.

### 2.7 Immunofluorescence staining

The skin tissues that were separated from the dorsal skin were preserved for an overnight period at 4 C using 4% paraformaldehyde (Sorlabio, Beijing, China). The fixed tissues were then subsequently treated with xylene for 30 min after being dried in an ethanol solution the following morning. Samples were incubated in xylene before being embedded in paraffin blocks. Using a Leica RM2255 microtome (Leica, Nussloch, Germany), the embedded paraffin blocks were cut to a thickness of 5–7 μm. The samples were then transferred to APES (ZSGB-BIO, Beijing, China) and treated with slides to prevent detachment.

For immunofluorescence staining analysis, the slides were deparaffinized in 100% xylene solutions for 30 min and then hydrated with an ethanol series. To perform antigen retrieval, slides were incubated in boiled 0.01 M sodium citrate buffer (pH = 6.0) for 10 min and then cooled down to room temperature. Blocking was performed with 3% BSA and 10% donkey serum in 0.5 M Tris-HCl buffer for 30 min at room temperature, and slides were then incubated with primary antibodies at 4 C overnight. The next morning, the slides were further incubated with secondary antibodies at 37 C for 30 min. DAPI was used to stain nuclei, and the slides were mounted with an anti-fade mounting medium. Pictures were taken under LEICA TCS SP5 II confocal microscopy (Leica Microsystems GmbH, Wetzlar, Germany). For enzyme substrate-based immunohistochemistry, the slides were washed with 3% H2O2 for 10 min to block endogenous peroxidase activity before blocking, and DAB (ZSGB-BIO, Beijing, China) solution was used for chromogenic reaction.

## 3 Results

### 3.1 Identification of goat hair follicle cell types using single-cell transcriptomics

**FIGURE 1:**
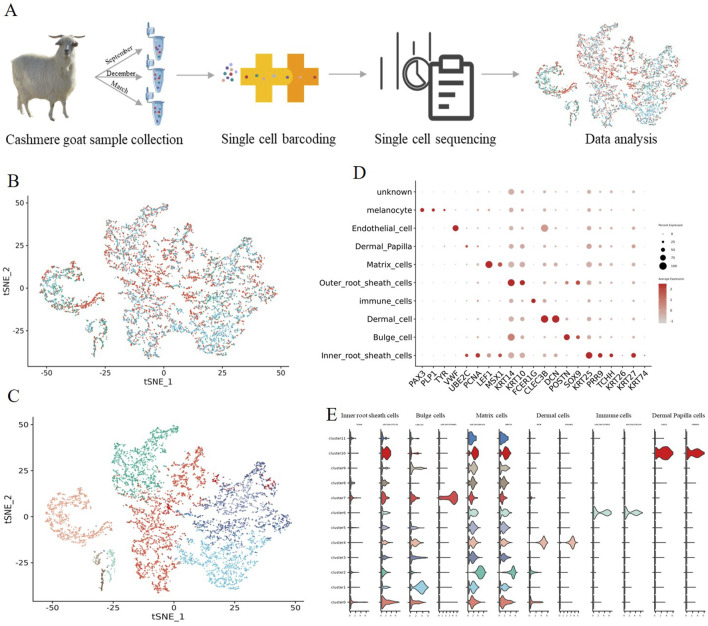
Identification of goat hair follicle cell types by single-cell RNA-seq transcriptomics. **(A)** Overall experimental design; **(B)** t-SNE plot of three periods in a single cell; **(C)** t-SNE plot of all single cells labelled. Different colors represent different cell clusters; **(D)** The dot plot shows distinct expression patterns of the selected signature genes for each cell type; **(E)** Expression specificity of the new marker genes in hair follicle cells.

To provide in-depth insight into the molecular profiles during cashmere goat hair follicle development and the main cell fate transitions, skin samples were collected from September (Anagen), December (Catagen), and March (Telogen) of the cashmere goat, and scRNA-seq was performed on the samples (Figure 1A). In total, 8,214 single cells were captured in three sample, and 20,140 genes、18,894 genes 、17,663 genes were detected for Anagen、Catagen、Telogen sample (Supplementary Table S1). We have carried out strict quality control, the proportion of mitochondrial genes (%) was retained: ≤20, the number of UMI identified in a single cell ≥100, and the number of genes identified in a single cell: 500–7,000 cells, after filtering, 19,705 single-cell transcriptome expression profiles were analyzed from the dorsal skin of Anagen (4,196 single cells), Catagen (2,773 single cells), and Telogen (724 single cells) cashmere goat fetuses (Supplementary Figure S1).

To investigate cellular heterogeneity, t-SNE analysis was performed, and 12 different cell clusters were identified across three developmental time points (Figures 1B,C), and different cell types were identified according to their marker gene expression (Supplementary Figure S2). Clusters 0 expressed high levels of the Inner root sheath cells lineage markers KRT25 and PRR9 (Takahashi et al., 2020), according to their marker gene expression; For clusters 1 and 7, they expressed high levels of the Bulge cell lineage markers POSTN (Joost et al., 2018), and they were termed as Bulge POSTN+; several important clusters were also identified including Matrix cells clusters (LEF1 and MSX1 clusters 2) (Ge et al., 2020; Schmidt-Ullrich et al., 2006); Outer root sheath cells cluster (KRT14 and KRT10, cluster 3 and 5) (Morita et al., 2021); Dermal cell cluster (CLEC3B and DCN, cluster 4); Immune cells cluster (FCER1G and laptm5, cluster 6); Endothelial cell cluster (VWF, cluster 8); Melanocyte cell cluster (PAX3 and PLP1, cluster 9) and Dermal Papilla cell cluster (UBE2C and PCNA, cluster 10) (Figure 1D) (Sardiña et al., 2019).

It is worth noting that a series of cell-type-specific expressed novel marker genes were identified in hair follicle cells of goats (Figure 1E). Such as Inner root sheath cells markers TCHH and LOC102177231, Bulge cell markers CXCL14 and LOC102176685, Matrix cells markers LOC102184223 and KRT35, Dermal cell markers COL6A1 and LUM, immune cell markers LOC102177855 and LOC102178129, and Dermal Papilla cell markers CRISP2 and GSG1. Collectively, we successfully identified different cell types at single-cell resolution and characterized their cell-type-specific gene expression patterns, and discovered novel markers for goat cell types, which provide potential biological markers for future research.

### 3.2 Gene expression signatures of hair follicle cells during apoptosis

**FIGURE 2:**
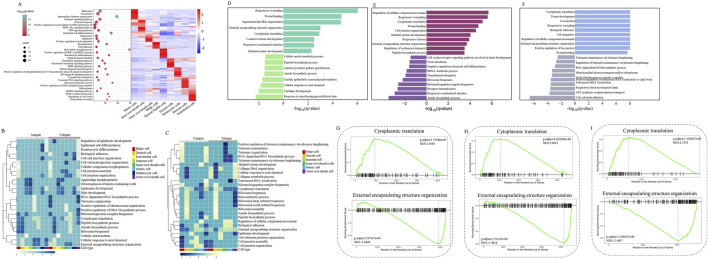
Gene expression signatures of hair follicle cells during apoptosis. **(A)** Right: heatmap showing the expression signatures of the expressed genes in each cell type; the value for each gene is the row-scaled Z score. Left: representative GO terms for specific genes; **(B)** A heatmap was visualized based on the highest enrichment DEGs between Anagen and Catagen hair follicle cells by GSEA; **(C)** A heatmap was visualized based on the highest enrichment DEGs between Catagen and Telogen goats hair follicle cells by the GSEA; **(D)** The histogram showing the biological process terms from GSEA in Anagen hair follicle matrix cells; **(E)** The histogram showing the biological process terms from GSEA in Catagen hair follicle matrix cells; **(F)** The histogram showing the biological process terms from GSEA in Telogen hair follicle matrix cells; **(G)** The trends of biological terms were obtained by GSEA in Anagen hair follicle matrix cells; **(H)** The trends of biological terms were obtained by GSEA in Catagen hair follicle matrix cells; **(I)** The trends of biological terms were obtained by GSEA in Telogen hair follicle matrix cells.

After identifying major cell types, we then investigated the molecular changes at single-cell resolution during hair follicle apoptosis. As shown in Figure 2A, GO analysis of the genes in each developmental stage (Anagen, Catagen, and Telogen) produced functions of nine major cell types, revealing the unique characteristics of hair follicle cells. GO terms specific to Inner root sheath cells included“regulation of cell migration”, “nucleus”, and “structural constituent of chromatin”, suggesting that aging-associated cell migration may be associated with nuclear maturation of Inner root sheath cells; GO terms including “positive regulation of DNA-templated transcription”, “extracellular exosome”, and “identical protein binding” were enriched for Bulge cells; GO terms including“iron ion transport”, “Wnt signaling pathway”, and “Apoptosis” for Matrix cells indicated that it plays an important role in the process of hair follicle cell apoptosis; GO terms including “Estrogen signaling pathway”, “protein kinase binding”, and “intermediate filament organization” for Outer root sheath cells; Dermal cells tend to be involved in the “Protein digestion and absorption”, “ECM-receptor interaction”, and “translation”; GO terms including “immune response”, “regulation of cell cycle”, and “MAPK signaling pathway” for Immune cells; Endothelial cells are mainly involved in “chemokine-mediated signaling pathway”, “NF-kappa B signaling pathway,” and“Chemokine signaling pathway”; GO terms including “Estrogen signaling pathway”, “melanosome”, and “anatomical structure development” for Melanocyte cells; Dermal Papilla cells tend to be involved in the “translation”, “structural constituent of ribosome”, and “Ribosome”. Collectively, GO enrichment shows that each cell type of the hair follicle (HF) is involved in a unique biological process.

To reveal the changes in gene expression signatures of cell types during HF apoptosis, following GSEA, a heatmap was visualized based on the highest enrichment DEGs per cell type from Anagen, Catagen, and Telogen apoptotic goat HF. Some important biological processes, such as“Biological adhesion” and “Cell junction organization”, were upregulated in the Bulge cells and Outer root sheath cells of Catagen compared with Anagen and Telogen, whereas these gene expressions were downregulated in Inner root sheath cells (Figures 2B,C). Compared with Anagen, “Ribonucleoprotein complex biogenesis” and “Ribosome biogenesis” were downregulated in other cell types of Catagen, but not in Matrix cells. It is worth noting that development-related “External encapsulating structure organization” and “Peptide biosynthetic process” are downregulated in most HF cells. Moreover, “Cytoplasmic translation”, “RNA-dependent DNA biosynthetic process”, and “Telomere organization” were decreased in Catagen, suggesting that apoptosis causes a decline in the developmental function of HF cells. To analyse gene-expression changes involved in apoptosis during the periodic growth and development of hair follicles, we identified biological processes of MCs in comparisons between different stages. Compared with Anagen and Telogen, the biological processes involved in “Response to mechanical stimulus” were downregulated, whereas “Peptide biosynthetic process” was upregulated in Catagen MCs (Figures 2D–F). GSEA also highlighted the most significant negative enrichment for genes of MCs upregulated in “‌Cytoplasmic translation” and positive enrichment for genes downregulated in “Regulation of cellular Component Movement” in Catagen compared with those of Anagen and Telogen. The changes in the gene expression signatures of MCs revealed that “Cytoplasmic translation” signaling was inhibited, whereas “Regulation of cellular Component Movement” was activated in hair follicle aging, suggesting that hair follicle aging is closely related to the stagnation of MCs proliferation and differentiation, cell migration, and cellular immunity.

### 3.3 The fate of matrix cells during HF apoptosis

**FIGURE 3:**
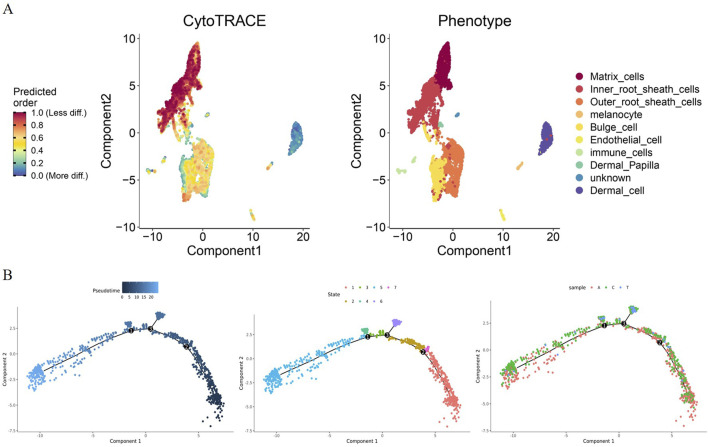
ScRNA-seq reveals the fate of MCs during apoptosis. **(A)** The differentiation potential of hair follicle cells was visualized by CytoTRACE analysis. The differentiation capacity from less to more is indicated by a gradient colour from red to blue; **(B)** Scatterplot showing the differential trajectories of MCs in Anagen, Catagen, and Telogen goat hair follicles with a pseudotime scale by Monocle analysis.

In the CytoTRACE analysis, the differentiation potential of HF cells was visualized with t-SNE. As shown in the plot, some HF cell types had a low degree of differentiation, such as Inner root sheath cells, Outer root sheath cells, Matrix cells, and Melanocytes, whereas Dermal Papilla, immune cells, and Dermal cells showed high differentiation potential, indicating that they are more functionally specific (Figure 3A). To reveal the temporal dynamics of MCs during apoptosis, the development trajectories were constructed by Monocle analysis, the pseudotime trajectory displayed three branch points, and the results clearly demonstrated the nonuniform development of MCs from Anagen to Telogen (Figure 3B). It is worth noting that most of the MCs in Anagen and Catagen goats were present in states 1,2,3,5, and most of the MCs in Telogen goats were present in states four and six (Figure 3B). These MCs showed a time-ordered decrease over pseudotime, indicating that the differentiation potential of MCs is gradually lost during apoptosis.

### 3.4 Reconstruction of the temporal dynamics of MCs during HF apoptosis

**FIGURE 4:**
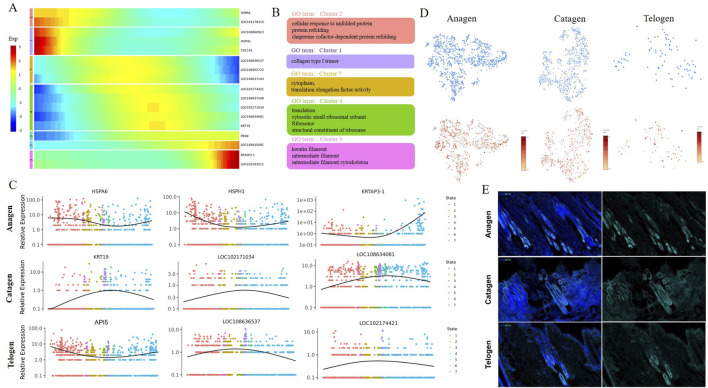
Pseudotime trajectory analysis delineated the temporal dynamics of MCs during hair follicle apoptosis. **(A)** Pseudotime heatmap showing dynamic gene expression profiles during MC fate commitment. The four gene sets were determined by k-means clustering according to their expression patterns. The expression level of dynamic genes from high to low is indicated by a colour gradient from red to blue; **(B)** The enriched GO terms for each gene set are shown based on the dynamic genes of GCs. The corresponding clusters’ GO terms are represented in the same colour; **(C)** Dynamic genes of expression trends of the signature genes over pseudotime in GCs; **(D)** Expression of API5 in MCs. The colour indicates the level of expression; **(E)** Immunostaining of hair follicles for API5.

After delineation of the trajectory inference, we focused on the temporal dynamics of MCs to reveal the changes in fate decisions during HF apoptosis. The trends of pseudotime-dependent genes along the pseudotime timeline were classified into seven clusters with different expression dynamics in a heatmap. As shown in the heatmap, the genes in Clusters 1,2,3, and five appeared to be upregulated along the pseudotimeline axis, and genes in Clusters 4,6, and seven showed the opposite trend (Figure 4A). Gene functional enrichment analysis revealed that Cluster 1 genes were highly enriched in the GO term “collagen type I trimer” (Figure 4B); Cluster two genes (such as HSPA6 and LOC102178315) were related to “cellular response to unfolded protein”, suggesting that these genes play important roles in the Protein synthesis process (Figures 4A,B); In addition, we also identified several genes, such as KRTAP3-1 and LOC102183211, which are involved in multiple processes, including“keratin filament” and “intermediate filament” in Cluster 3 (Figures 4A,B), specifically, KRTAP3-1, the keratin-associated protein, is important for cashmere fiber structure (Bartling et al., 2019), and its expression showed an obvious upward trend along the pseudotimeline axis (Figure 4A); GO analysis enriched the generation of “translation” and “structural constituent of ribosome” in Cluster 4 (Figure 4B), the activity of those biological processes was also inhibited along the pseudotimeline axis, indicating that HF development gradually weakened.

Subsequently, we then analysed the gene dynamics of MCs at different stages. As expected, a series of dynamic genes (HSPA6, HSPH1, and KRTAP3-1) of MCs showed decreases first and then increased expression over pseudotime in Anagen (Figure 4C). These dynamic genes play an important role in the process of cell proliferation, differentiation, and apoptosis. Some specific genes involved in cell differentiation and translation, such as KRT19, LOC102171034, and LOC108634081, are mainly enriched in Catagen (Figure 4C).On Telogen, LOC108636537 and LOC102174421 showed a trend of increasing first and then decreasing along the pseudotime axis and promote the GTP-dependent binding of aminoacyl-tRNA to the A-site of ribosomes during protein biosynthesis; API5 showed the opposite trend along the pseudotime axis (Figure 4C), API5 is known to be highly expressed in the Anagen period, but the expression level is reduced in the Catagen and Telogen (Figure 4D). We also observed that API5 was abundant in hair follicles by immunostaining, hardly expressed in the internal results of hair follicles during the Telogen and Catagen, while the expression of hair follicle roots during the Anagen is abundant (Figure 4E). In total, we recaptured the sequential and stepwise trajectory of MCs development and identified a series of pseudotime-dependent genes that may play a role in development in HF aging.

### 3.5 Transcriptional regulation of MCs during HF aging

**FIGURE 5:**
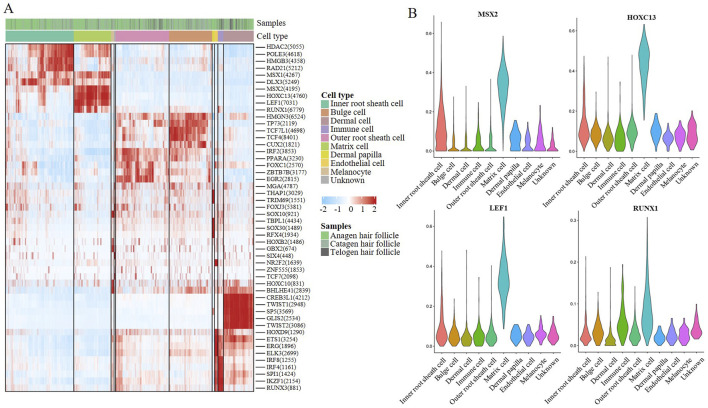
Transcriptional regulation of MCs during hair follicle apoptosis. **(A)** A heatmap visualized the significant regulons by SCENIC analysis in hair follicle cells. The score of regulation from high to low is indicated by a colour gradient from red to blue. The number in parentheses indicates the number of target genes regulated by this transcription factor; **(B)** Violin plot showing feature TF expression in hair follicle cells.

To further explore the regulatory factor of hair follicle apoptosis, we analysed the differentially expressed Transcription factors (TFs) in the HF of Anagen, Catagen, and Telogen. A heatmap of the AUC scores of TF motifs was visualized by SCENIC analysis in the main cell types. We identified 1,281 significant regulons that regulated hair follicle gene expression patterns (Additional file 2). It is worth noting that MSX2, HOXC13, LEF1, and RUNX1 motifs were highly activated in MCs (Figure 5A). MSX2 was found to be associated with embryonic morphogenesis; Hoxc13 can directly regulate the expression of keratin and keratin-associated proteins, and is a key influencing gene leading to hair removal in mice; similar to the previous GO enrichment in “positive regulation of granulocyte differentiation”, LEF1 and RUNX1 have profound roles in the transcriptional regulation. Subsequently, we compared the differential transcription factors for hair follicle cell types, we found that transcription factor genes such as MSX2, HOXC13, LEF1, and RUNX1 were highly expressed in MCs (Figure 5B). Based on SCENIC analysis of hair follicle in different stage groups, the transcription factors showed that MSX2, HOXC13, LEF1, and RUNX1 expression levels did change with time in MCs, these results suggest that the hair follicle development stages show different gene expression patterns, and MSX2, HOXC13, LEF1, and RUNX1 may play a key role in the apoptosis process.

## 4 Discussion

In this study, we present the first single-cell survey of HF apoptosis in goats that provides new insights into the mechanisms by which transcriptional profiles change during apoptosis. Different cell types have been identified in the human, mouse, and sheep adult skin, including Endothelial cells, Mesenchymal cells, Immune cells, and Melanocytes (Takahashi et al., 2020; Joost et al., 2018; Ge et al., 2020). In the present study, nine main cell types were successfully identified, including Inner root sheath cells, Bulge cells, Matrix cells, Outer root sheath cells, Dermal cells, Immune cells, Endothelial cells, Melanocyte cells, and Dermal Papilla cells. Consistent with previous reports, marker genes such as KRT25, KRT14, FCER1G, LEF1, and MSX1were specifically expressed at high levels in Inner root sheath cells, Outer root sheath cells, immune cells, and Matrix cells (Schmidt-Ullrich et al., 2006; Morita et al., 2021; Sardiña et al., 2019; Bartling et al., 2019). However, some markers, such as S100a4, Lhx2, and Wif1, in immune cells, ORS, and Dermal cells of mice or humans, were not found in goat hair follicles, suggesting that the marker genes in goat ovaries are not completely conserved with those in other animals (Rendl et al., 2005; Nakakura et al., 2021). Subsequently, we identified a series of cell-type-specific expressed novel marker genes, including TCHH, CXCL14, KRT35, GSG1, CRISP2, LOC102177231, LOC102176685, LOC102184223, LOC102177855, and LOC102178129, which provided valuable information to distinguish HF cell types of other species.

Most previous reports have described detailed morphological alterations during HF apoptosis in goat, such as periodic transformation of hair follicles, whereas the molecular mechanisms underlying HF apoptosis remain largely unknown at the single-cell level. Here, we show that HF cell types are involved in their own unique biological processes by using scRNA-seq. For example, the Wnt signaling pathway was enriched in Matrix cells, and MCs were involved in cell apoptosis. It is well accepted that Wnt signalling is essential to the initiation of hair growth and transmits signals among many types of stem cells during the formation of hair placodes (Zhang et al., 2009). In addition, the comparative analysis of each stage of the hair cycle revealed that the apoptosis process of hair follicle cells was accompanied by significant changes in the expression of the Wnt signaling pathway and apoptosis-related genes in MCs. The regulation of HF apoptosis is a cross-species conserved complex process involving multiple signaling pathways and key molecules. In mice, B-cell lymphoma-2 (BCL-2) family proteins are key regulators of HFs' apoptosis. The anti-apoptotic protein MCL-1 is essential for the survival of activated hair follicle stem cells (HFSCs), and its deletion triggers apoptosis and ablation of hair regeneration by activating BAK and P53 signaling pathways (Ankawa et al., 2021). On the contrary, the pro-apoptotic protein BIM is upregulated during degeneration and promotes cell death (Yang et al., 2022). In addition to the BCL-2 family, the TGF-β pathway has a dual role in HF cell fate determination. Although TGF-β can promote cell proliferation to a certain extent, excessive TGF-β signaling can drive apoptosis and promote cells to enter the degenerative phase (Dinh and Wang, 2022). Another key pathway is the EDA-EDAR-NF-κB axis. Mutations in EDA or its receptor EDAR can lead to impaired HF development and reduced appendages. This pathway interacts with Wnt signaling and regulates apoptosis during degeneration (Song et al., 2019). Supporting these findings, previous studies revealed that keratinocyte differentiation, epidermis development, and skin epidermis development were observed in MCs through scRNA-seq analysis (Ge et al., 2020). MCs play a role in the cyclical growth and development of hair follicles. We found that MCs were less differentiated by CytoTRACE analysis, suggesting that the morphological structure and function of MCs may be more complex. Based on single-cell pseudotime trajectory inference, the number of Matrix cells is gradually depleted with month, especially in Catagen, which are almost depleted. By analysing the expression of different genes over pseudotime, we identified a series of dynamic genes involved in hair follicle function. These dynamic genes were enriched in multiple biological processes, including the collagen type I trimer, cellular response to unfolded protein, keratin filament, intermediate filament, translation, and structural constituent of ribosome. Ge et al. (2020) reported that subpopulations of MCs play distinct roles in hair follicle development. Interestingly, we found that dynamic genes, such as KRTAP3-1, it is well known to KRTAP3-1 belong to the KAP3 gene family, which is the smallest in high-sulfur KAPs and is very important in the structure of hair follicle products (Rogers et al., 2001). Similarly, MC functional genes, such as KRT19、 HSPA6 、 HSPH1 、LOC102171034、LOC108634081, and API5 also showed distinct expression characteristics in subcluster MCs in this study. This evidence suggests that the fate and functions of subcluster MCs are inconsistent, and the distinct roles are largely determined by their gene expression patterns during hair follicle apoptosis. Specifically, KRT19、LOC102171034, and LOC108634081 showed similar trends over pseudotime and were highly expressed in MCs, indicating that these genes play a coordinating role in hair follicle development and apoptosis. It is well known that KRT19 plays vital roles in the balance between proliferation and differentiation during the HF cycle (Taha et al., 2025; Sharma et al., 2024). HSPA 6 may play a dual role in cell homeostasis and stress response, and API5 anti-hair follicle cell apoptosis (Zhang et al., 2024; Choi et al., 2024; Deng et al., 2024; Bong et al., 2024; Matsuzawa-Ishimoto et al., 2024). This study provides insights into the molecular mechanism of apoptosis behind hair follicle cycle transformation.

## 5 Conclusion

In summary, the present study provides a comprehensive single-cell transcriptomic map of Anagen, Catagen, and Telogen goat hair follicles and broadens our understanding of cell identities and growth cycle gene-expression alterations in MCs. For the first time, the study distinguished differences in the developmental trajectories and expression patterns of subcluster MCs in goats. Importantly, this study revealed the molecular mechanism of hair follicle apoptosis in goats and provided scientific support for the sustainable development of the goat industry. In addition, we identified a series of important genes or signalling pathways associated with hair follicle apoptosis at the single cell level, such as KRT19, API5, LEF1, and Wnt signaling pathways, providing new targets for improving hair follicle period transfer in goats.

## Data Availability

The raw data supporting the conclusions of this article will be made available by the authors, without undue reservation.
