# Supplementary material for: A single-cell transcriptomic atlas elucidates the hair cycle and apoptosis mechanisms in goat hair follicles
Source: Front Cell Dev Biol. 2025 Nov 5;13:1693637. doi: 10.3389/fcell.2025.1693637 (PMC12627004; doi:10.3389/fcell.2025.1693637)
Supplement: Supplementary file 1 [file DataSheet1.docx]

Supplementary Material

# Supplementary Figures and Tables

## Supplementary Figures


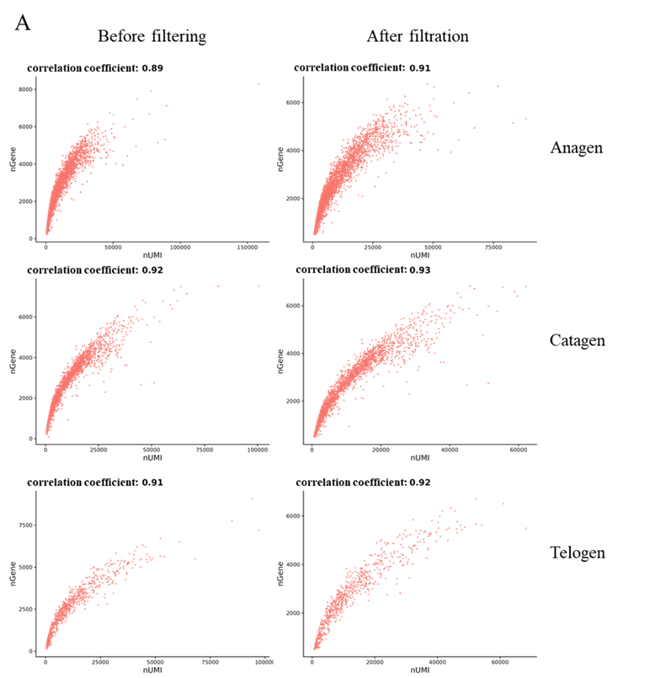


**Supplementary Figure 1.** Scatter plot of each sample cell before and after filtration


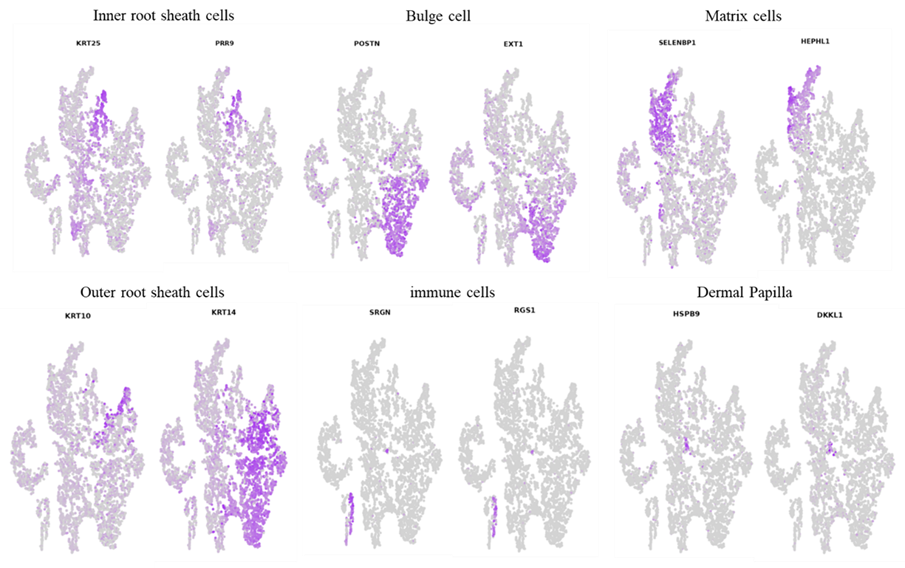


**Supplementary Figure 2.** The t-SNE distribution of different cluster maker genes.

## Supplementary Tables

| Sample information | Anagen | Catagen | Telogen |
| --- | --- | --- | --- |
| Estimated Number of Cells | 4541 | 2859 | 814 |
| Median UMI Counts per Cell | 5142 | 5837 | 8515 |
| Mean Reads per Cell | 111301 | 178551 | 714085 |
| Median Genes per Cell | 1971 | 2096 | 2,440 |
| Total Genes Detected | 20140 | 18894 | 17663 |

**Supplementary** Table 1. CellRanger Analysis Cell Information Statistics
